# At the earliest: a Hub and Spoke referral and referral-back pilot project increases access to liver transplantation and ensures good long-term care

**DOI:** 10.1007/s13304-025-02262-6

**Published:** 2025-05-26

**Authors:** Cecilia Pravadelli, Alberto Ferrarese, Luisa Moser, Francesco Paolo Russo, Giacomo Germani, Marco Senzolo, Martina Gambato, Alberto Zanetto, Sara Battistella, Elisa Menotti, Flora Agugiaro, Giovanni Vettori, Giovanni de Pretis, Pamela Ballotta, Ivana Maioli, Armando Gabbrielli, Lucia Pilati, Tiziano Martello, Umberto Cillo, Patrizia Burra

**Affiliations:** 1 Gastroenterology, Trento Hospital, Trento, Italy; 2 Multivisceral Transplant Unit, Gastroenterology, Padua University Hospital, Via Giustiniani 2, 35122 Padua, Italy; 3 Department of Surgery, Oncology, Gastroenterology, Padua University Hospital, Via Giustiniani 2, 35122 Padua, Italy; 4 General Surgery 2 and Liver Transplant Unit, Padua University Hospital, Padua, Italy; 5 Medical Management Unit, Padua University Hospital, Padua, Italy; 6 Intensive Care Unit, Trento Hospital, Trento, Italy; 7 Center for Medical Science (CISMed), University of Trento, Trento, Italy

**Keywords:** Cirrhosis, Quality of life, Acute-on-chronic liver failure

## Abstract

Liver transplantation (LT) represents an effective therapeutic option for patients with end-stage liver disease and hepatocellular carcinoma. However, barriers to access LT programs still exist for many patients, including distance from transplant centers and delays in referral. Furthermore, long-term care is required also in stable LT recipients. This pilot study aims to evaluate the characteristics and outcomes of patients with end-stage liver disease referred to Padua University Hospital following the implementation of a structured referral program with Trento Hospital. Furthermore, the study assesses the outcomes of LT recipients who were referred back and continued follow-up care at the Spoke Center. After an internal work reorganization at the Spoke Center to improve care for patients with liver disease, we designed this prospective pilot study based on a structured referral and referral-back program for managing patients before and after LT. Accordingly, all inpatients potentially eligible for LT were transferred from the Gastroenterology Unit at Trento Hospital to the Multivisceral Transplant Unit at Padua University Hospital between 2020–2023. Similarly, stable LT recipients were referred back to the Spoke Center for management of long-term follow-up. During the study period, 27 adult inpatients (59% male; median age 50 [42–51] years) deemed eligible for LT were consecutively referred from Trento to Padua. The median [IQR] MELD score at the time of referral was 26 [23–30], and the length of stay at the LT Center was 21 [18–23] days. At the end of follow-up, 6 (22%) patients underwent LT, while the transplant-free survival rate was 37%. During the same period, 27 stable adult LT recipients (55% male; median age at referral back 56 years; median time since LT 9 years) living near Trento were referred back to the Spoke Center. During the follow-up, n.19 (70%) patients experienced at least one medical complication (40% liver-related), the majority of these being managed at the Spoke Center. LT recipient satisfaction of this way of care Center was high. A structured Hub and Spoke collaboration increase access to LT, making it more equitable, and improves the management of stable LT recipients closer to home.

**Graphical abstract:**

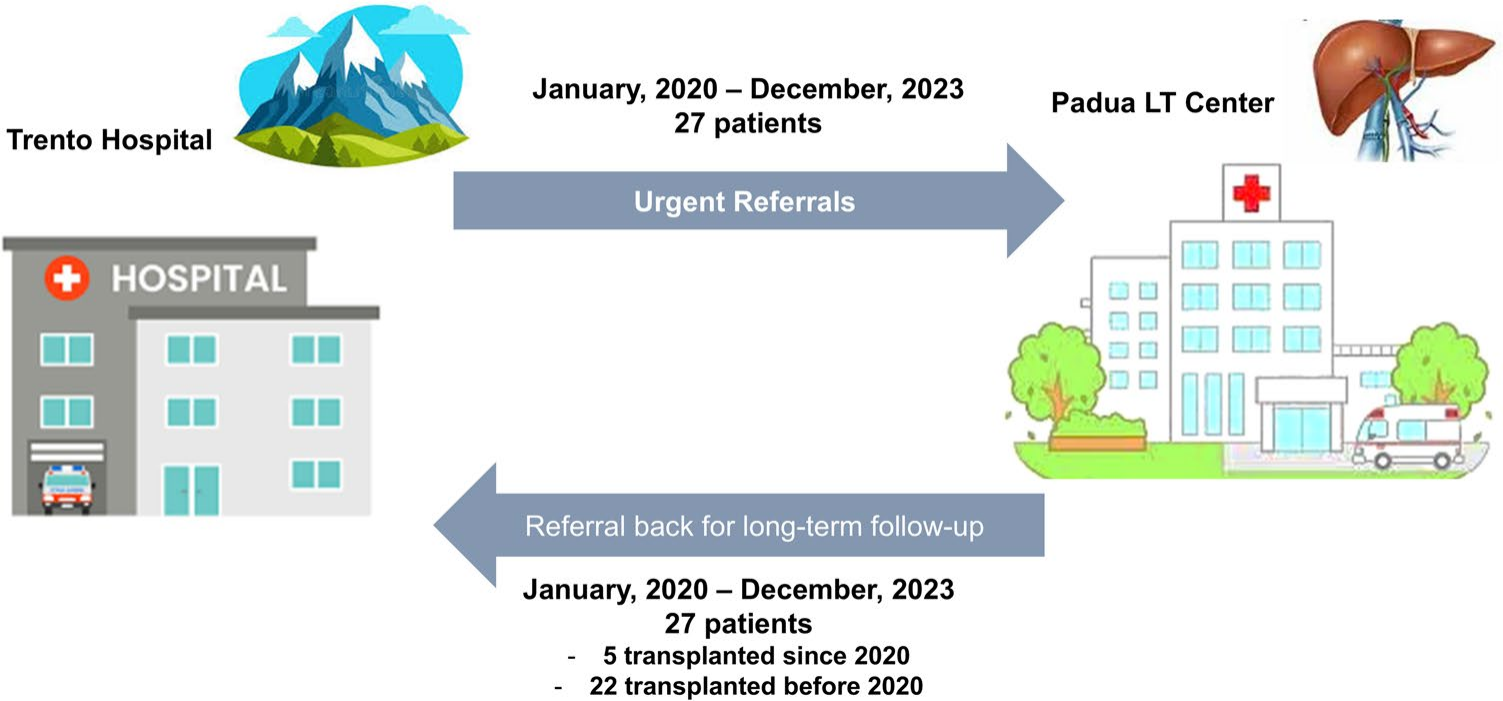

## Introduction

Liver transplantation (LT) is a therapeutic option for selected patients with end-stage liver disease (ESLD) and hepatocellular carcinoma (HCC), offering a survival benefit compared to other available treatments. Over time, the criteria and indications for LT have expanded, allowing more patients to undergo transplant evaluations than in the past [1, 2]. A prompt referral to the LT center from neighboring hospitals is crucial to ensure early access to care and equity among patients. Late referrals, indeed, can reduce the chances of transplantation due to deteriorating health conditions and/or clinical complications that may delay transplant evaluation [3–5]. The Hub and Spoke network model, based on a bidirectional flow between Referring Hospitals and LT Center, has shown increased access to transplantation and reduced geographical disparities both in Italy and in other Countries [6, 7]. Furthermore, the wise expansion of transplant criteria, along with increased donor availability, has led to a significant rise in the number of transplants performed each year in Italy. Since all LT recipients require personalized follow-up care after surgery, for the management of surgical and medical complications, strategies to prevent bottlenecks at tertiary centers must be implemented at various levels. One such strategy could be the proposed “referral back” model, in which stable post-LT patients could continue their follow-up at first- or second-level hospitals.

Trentino is a region in Northern Italy, located near the Alps, with a population of 550,000 inhabitants. It has limited rail transport and lacks an airport network. There is no LT program in place, so potential candidates are referred to neighboring transplant centers. In the past, the referral process for patients living in the Trentino region was not well-structured, allowing patients to choose from three different transplant centers based on their preferences and transplant eligibility. Additionally, Trento Hospital, a 750-bed facility with intensive care, gastroenterology, digestive endoscopy, and general surgery units, have not still developed a structured a network capable of managing post-LT medical complications.

Given these challenges, a pilot project was launched in 2020 at the Gastroenterology Unit, Trento Hospital. An internal reorganization was initiated with the aim to improve care for patients with liver disease, both before and after LT. Several innovations were introduced, such as centralization of second-level endoscopy services; creation of specialized in- and out-patient medical teams dedicated to LT candidates and recipients, led by two transplant hepatologists; empowerment of an inter-hospital working group with the Padua Liver Transplant Center during specific meetings host at Padua University.

Therefore, this study aims to describe the results of this pilot project involving Gastroenterology Unit at Trento Hospital and the of Padua LT Center. In detail, we aimed to describe:

- The characteristics and outcomes of patients with ESLD referred to Padua University Hospital after introduction of this structured referral program.
- The characteristics and outcome of LT recipients who were referred back and continued the follow-up at the Spoke Center.
- The impact of this project on the activity at Gastroenterology Unit, Trento and on patient’s satisfaction.

## Materials and methods

The study has an observational, prospective design and involves two centers: Gastroenterology at Trento Hospital and Multivisceral Transplant Unit, at Padua University Hospital. Patients’ data were collected on a shared, prospectively updated, dataset. The project has been approved by the Local Ethical Committee (protocol code: TXHS).

A shared database containing information about patient referrals and follow-up was created. Patients were categorized based on transplant outcomes as either uncomplicated or complicated. It was decided that uncomplicated patients would be progressively managed by the Spoke Center, while patients with unmanageable complications at the Spoke Center would have prompt access to the Hub Center. Once complications were resolved, these patients resumed follow-up care primarily at the Spoke Center. Bimonthly telematic meetings between the two medical teams were held to carefully discuss critical medical aspects and present new transplant cases. New data and information were regularly updated in the database.

### Inclusion criteria

#### Referred patients

Consecutive adult patients hospitalized at Gastroenterology, Trento Hospital, between January 1, 2020, and December 31, 2023, who were potentially eligible for LT evaluation (acute decompensation, acute-on-chronic liver failure, acute liver injury) were included. Outpatients were excluded due to their heterogeneous clinical characteristics and indications for LT. The diagnosis of liver cirrhosis was based on clinical, radiological, and biochemical criteria, while HCC was diagnosed according to guidelines using radiological and/or histological criteria. Although there was no strict age limit that would automatically exclude a patient from being considered for transplant evaluation, patients over 75 years of age were generally not considered as transplant candidates. Each case was discussed with the Transplant Center through a dedicated referral form. Patients deemed potentially eligible for LT were sent to the Transplant Center for further evaluation. During meetings between two centers, each case was discussed, allowing for possible referral back before transplantation if the patient no longer met the criteria for LT or if his/her condition became too severe to proceed with LT. Patient outcome was recorded at time of clinically relevant events (e.g., death or transplantation), or within 3 months after referral.

#### Referred back patients

Since 2020, the Hub Center started referring back to Gastroenterology Unit, at Trento Hospital all LT patients living in Trentino Region who achieved clinical stability. This group of patients included not only those who had recently undergone LT (since 2020), but also those who, despite residing in the Trentino Region, had previously continued the follow-up at the LT Center. Considering patients who had undergone LT since 2020, the Hub and Spoke centers agreed that the former would manage the patient's care within the first two years after the transplant. After this period, the Gastroenterology Unit at Trento Hospital would take over the patient's follow-up, with updates provided through quarterly inter-hospital meetings. The spoke center became responsible for managing immunosuppressive therapy, metabolic complications, and surveillance for de novo neoplasms. A thorough discussion between Two Centers was needed in case of vascular, biliary, and immunological complications, as well as the management of de novo cancers. Endoscopic treatment of biliary complications could be handled at the spoke center following a shared clinical plan, while endoscopic or surgical treatment of refractory biliary strictures should be carried out at the LT Center. We used two simple indicators to assess the potential impact of this pilot project on overall activity at Gastroenterology Unit, Trento Hospital: (a) the number of outpatient visits for LT patients by year; (b) the number of endoscopic retrograde cholangiopancreatographies (ERCPs) performed for anastomotic biliary stricture in LT recipients.

We also tested LT patients’ satisfaction and the impact on everyday life through the questionnaire proposed by Tai et al., [6] which was translated into Italian language and adapted, accordingly. This questionnaire was completed by patients during outpatient clinic visits at Trento University Hospital, at least one year after referral back.

### Statistical analysis

Dichotomous variables and continuous variables were reported as percentages and medians [IQR], respectively. The comparison between dichotomous variables was performed using chi-square test, whereas the comparison between continuous variables was performed using the Mann–Whitney *U* test. A difference was considered statistically significant when *p* < 0.05. The transplant free survival (TSF) analysis was conducted using Kaplan–Meier curves; curves were compared using the log-rank test.

## Results

### Referral from trento to Padua Hospital

**Fig. 1:**
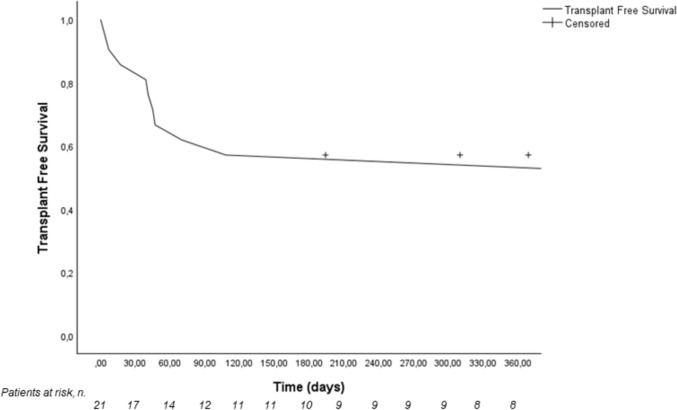
Cumulative transplant free survival of in-patients urgently referred from Trento Hospital to Padua liver transplant Center

**Fig. 2:**
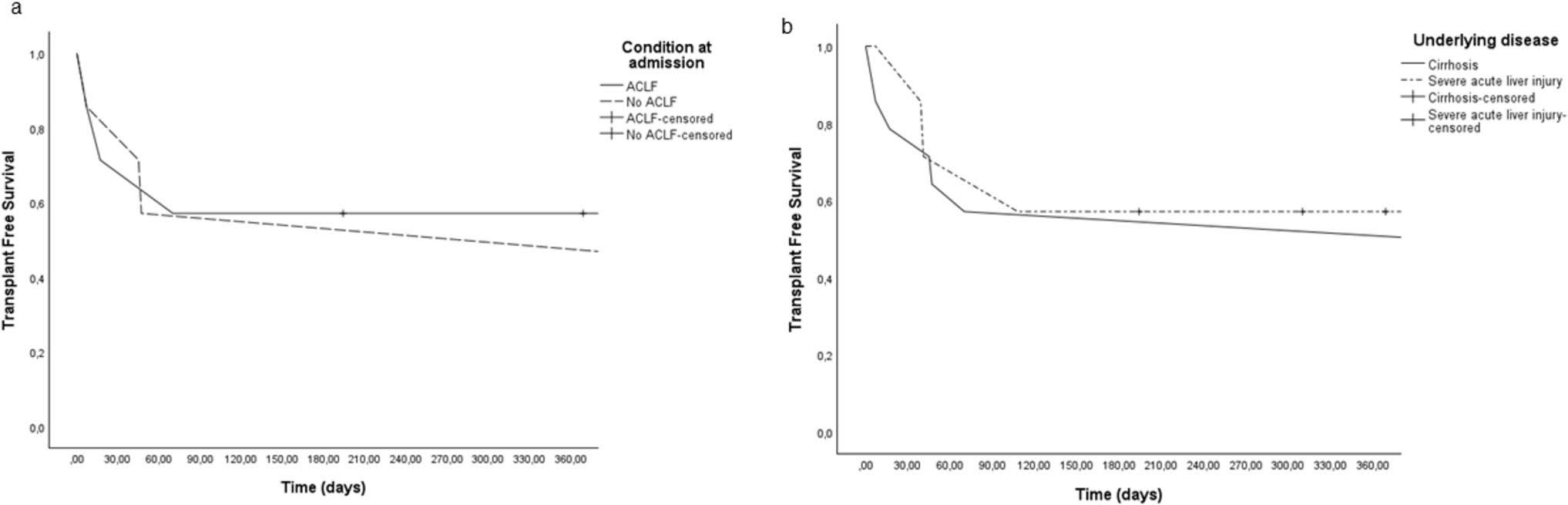
Transplant free survival of in-patients urgently referred from Trento Hospital to Padua liver transplant Center, according to the underlying condition; **a** presence of acute-on-chronic liver failure; **b** cirrhosis vs. severe acute hepatitis

From January 2020 to December 2023, 27 patients were referred from the Gastroenterology, at Trento Hospital to Multivisceral Transplant Unit, Padua LT Center (Table 1). Among these, 18 (66.7%) were referred for ESLD and 9 (33.3%) for severe acute hepatitis. Considering ESLD, alcohol represent by far the most common etiology (15/18, 83.3%), whereas 2/18 (11.1%) and 1/18 (5.6%) patients had an autoimmune hepatitis and primary sclerosing cholangitis, respectively. The median (IQR) MELD and MELD-Na scores at time of referral were 26 (23–30) and 28 (24–32), respectively; more than half of patients (10/18, 55%) fulfilled the criteria for ACLF at the time of referral. Considering patients severe acute hepatitis, drug-induced liver injury and autoimmune hepatitis and represented the most common underlying disease (44.4% and 22.2%, respectively). The median (IQR) length of stay at Padua LT center, calculated at time of death, LT or patient discharge was 21 (18–23) days. During their first admission, *n*. 2 (7%) patients underwent LT whereas n.4 (14%) died. At the end of follow up, 10 (37%) were alive without LT, 6 (22.2%) had undergone LT and 11 (40.8%) died. Considering the whole cohort of patients, the transplant free survival at 30, 60, 90 days after admission at Padua LT Center was 80%, 69% and 57% respectively (Fig. 1). There was no difference in the transplant free survival at 30, 60, 90 days in the group of patients referred for ESLD according to presence or absence of ACLF at the time of referral (*p* = 0.84, log-rank test, Fig. 2a) and when comparing the group of patients referred for ESLD to the group referred for severe acute liver injury (Fig. 2b).

**Table 1:** Clinical and demographic features of in-patients referred from Gastroenterology at Trento Hospital to Padua LT Center in the study period

PATIENTS	*n*. 27
Male, *n*. (%)	16 (59)
Age (median, IQR)	50 [42–51]
Underlying disease, *n*. (%) End-stage liver disease Alcohol Autoimmune Cholestatic Severe acute hepatitis Drug-induced liver injury Autoimmune hepatitis Viral Unknown etiology	18 (66.7) 15 (55.6) 2 (7.4) 1 (3.7) 9 (33.3) 4 (14.8) 2 (7.4) 1 (3.7) 2 (7.4)
Scores at time of referral MELD score MELD-Na score	26 [23–30] 28 [24–32]
ACLF, *n*. (%)*	10 (55.5)
Hospital length of stay, days	21 [18–23]
In-hospital outcome, *n*. (%) Alive Dead Liver transplantation	21 (77.8) 4 (14.8) 2 (7.4)
Final outcome, *n*. (%) Alive Dead Liver transplantation	10 (37) 11 (40.8) 6 (22.2)

^*^considering only patients with end-stage liver disease. *ACLF*: Acute-on-chronic liver failure; *MELD*: Model for end-stage liver disease

### Referral back from Padua University Hospital to Trento Hospital

**Fig. 3:**
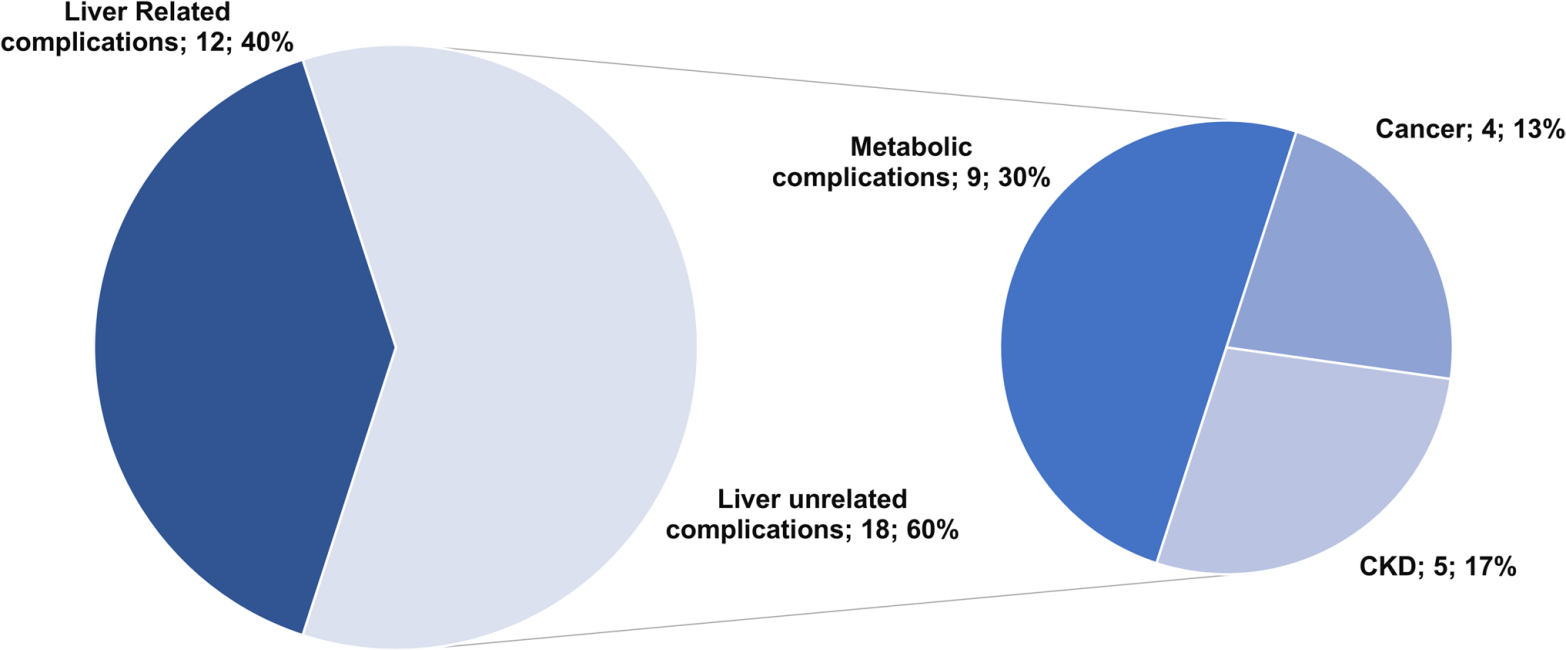
Post-transplant medical complications occurred in patients referred back to Trento Hospital. *CKD*: chronic kidney disease

A total of 27 patients living in Trentino Region who had undergone LT at Padua University Hospital were referred back to Trento Hospital starting in 2020 (Table 2). The majority of patients (55.5%) were male and the main indications for LT was viral (37%), alcoholic (22.2%), cholestatic (18.5%). Figure 3 showed the most important complications experienced after referral back. At least one medical complication occurred in 19 (70%) cases. Graft related complications accounted for 40% overall complications, being anastomotic biliary stricture (6 cases, 22%) the most frequent event, followed by vascular complications (4 cases, 14.8%) and chronic rejection (7.4%). All patients with biliary stricture underwent ERCP with multi-stenting (a median value of 4 procedures per patient); achieving liver enzymes normalization and complete resolution of anastomotic stricture at subsequent radiological evaluations. None of the patients needed surgical approach. Considering liver-unrelated complications, there were *n*. 5 cases of post-LT chronic kidney disease, whereas n. 9 new diagnoses of diabetes, hypertension and dyslipidemia occurred. All patients except one who experienced cardiovascular and metabolic events were transplanted before 2020. De novo cancer occurred in 4 patients (one breast cancer, one bladder cancer, one lymphoma and one leukemia). After a median follow-up of 32 [22–41] months, one patient was lost at follow up, one patient was retransplanted due to late hepatic artery thrombosis, and one patient died of cancer.

**Table 2:** Clinical and demographic features of liver transplant recipients referred back from Padua University Hospital to Trento Hospital in the study period

PATIENTS	*n*. 27
Male, *n*. (%)	15 (55.5%)
Age (median, [IQR])	56 [17.5]
Time from liver transplantion at the beginning of referral back, years [IQR]	9 [14]
Etiology of liver disease, *n*. (%) Alcohol Viral Autoimmune Cholestatic Others	6 (22.2%) 10 (37%) 2 (7.4%) 5 (18.5%) 4 (14.%)
Immunosuppressants, *n*. (%) Tacrolimus monotherapy Tacrolimus + everolimus Tacrolimus + mycophenolic acid Cyclosporin monotherapy	12 (44.4%) 2 (7.4%) 9 (33.3%) 4 (14.9%)
Development of at least one medical complication after liver transplantation, *n* (%)	19 (70.4%)
Follow-up time at Spoke Center, months [IQR]	48 [37–51]
Outcome, *n*. (%) Alive Dead Lost at follow-up	25 (92.6%) 1 (3.7%) 1 (3.7%)

### Impact of the pilot project on gastroenterology at Trento Hospital and on patients’ satisfaction

We explored the impact of the referral back project on outpatient activity at Gastroenterology, Trento Hospital. We showed a significant increase of outpatients’ consultations for LT recipients (from 224 in calendar year 2019 to 359 in calendar year 2023, + 61%). Similarly, the number of ERCP on LT patients in Trento Hospital increased over time (from 9 in 2019 to 30 in 2023). Table 3 shows the satisfaction rate of patients for the four questions asked during outpatient visits at the Spoke Center. Notably, 88% of respondents rated the communication between the two centers as good, while in 80% of cases, management at the Spoke Center was not considered disadvantageous. The median satisfaction score [IQR] among respondents was 7 [6–8].

**Table 3:** Results of the satisfaction questionnaire of LT recipients who were referred back to the Spoke Center

Questions	Satisfaction rate *n*. (%)
• I think there is good collaboration between the Hub and Spoke Center. This may significantly improve the care of my condition.*	Agree: *n*. 23 (88.4%) Disagree: *n*. 3 (11.6%)
• I feel disadvantaged being treated at the Spoke Center.*	Agree: *n*. 5 (19.2%) Disagree: *n*. 21 (80.7%)
• The best advantage of being treated at the Spoke Center is:** Receiving care near home Time spared Reducing cost I prefer not to answer	17 (68) 4 (16) 2 (8) 2 (8)
• Describe the overall satisfaction rate in a scale from 0 to 10**	7 [6–8]^§^

^*^n.26 patients answered this question

^**^n.25 patients answered this question

^§^median [IQR]

## Discussion

This pilot project described for the first time the role of referral and referral back in patients who are potential candidates for LT and in those who have undergone LT, respectively, reporting the outcomes of close collaboration between the Trento Hospital and the Padua LT Center. Considering the characteristics of the referred patients, they are in line with the epidemiological trend observed in recent decades in Europe and in our country. In fact, there has been a significant decrease in patients with viral etiology, while alcohol-related cirrhosis represented the main etiology in patients with ESLD [8, 9]. It is also important to highlight that, at time of referral, many patients (55%) had ACLF, a potentially life-threatening condition portending high short-term mortality, especially when the access to LT program is delayed.

Evaluating the early outcomes of these patients, it is interesting to note that mortality was quite high (around 15%) and that 90-day TSF was roughly 50%. This reflects the severity of the transferred patients and underscores the need for early referral. According to Fig. 2a, there was no difference in transplant-free survival between patients with and without ACLF (*p* = 0.84, log-rank test). Possible explanations include the small sample size and the identification of ACLF at the time of transfer, rather than during the entire observation period. Additionally, the small number of patients with ACLF did not allow for proper stratification based on the grade of ACLF. Similarly, no difference in TSF has been found between patients with severe acute liver injury and ESLD (Fig. 2b), probably because of similar severity of both conditions.

The creation of these referral and referral back pilot projects present multiple advantages, both for healthcare facilities and for patients. Considering peripheral Hospitals, they can benefit from the early referral of patients with potential transplant indications, through a traceable and efficient method. Additionally, they can benefit from the referral back by gradually and progressively increasing their expertise in managing medical and endoscopic complications in LT recipients, being able to discuss complex cases (e.g., immune-mediated acute damage, vascular injuries) with the Transplant Center, and agreeing on the best therapeutic strategy. As for the Transplant Center, this project has the advantage of ensuring quick access to transplant resources for patients with ESLD, both having cirrhosis and severe acute liver injury. This aspect is particularly important in more severe patients, such as those with ACLF and/or ALF, where the prompt evaluation for LT is crucial to grant access to the transplant waiting list at the earliest. Moreover, improving the expertise of Peripheral Centers helps avoid a bottleneck effect, which, given the annual increase in the number of patients undergoing transplantation, would otherwise be inevitable. This methodology will likely need to be implemented in the future, along with greater involvement of General Practitioners, for the proper management of long-term medical complications in liver transplant recipients. [10]

The impact of this project has undoubtedly had positive effects on the overall management at the Spoke Center. It has increased the awareness of healthcare providers about liver transplantation and their willingness to consider patients as potential candidates for transplantation. These factors, together with logistical and organizational improvements and the expansion of transplant criteria, have led to a significant increase in the number of patients undergoing LT from Trento Hospital (+ 29% from 2017 to 2023, compared to the number of patients per year from 2009 to 2016). This hub-and-spoke model can subsequently be implemented in other care settings for patients with advanced and/or acute liver diseases, possibly including medium-volume centers as well.

It is also important to emphasize how this project can be highly beneficial for the patient: they benefit from receiving fair and quick access to care, regardless of their place of residence, and from receiving appropriate care, once stabilized, in the same hospital that had previously managed their case. Furthermore, especially during the pre-transplant phase, this project appears effective in ensuring equitable access to care, and specifically to the LT program, regardless of the distance to an LT Center. [11, 12]

This project was conceived before the COVID-19 pandemic. The pandemic also introduced various possibilities for accessing care, such as tele-consultation. This method can be potentially effective in ensuring fair access to care for an ever-increasing number of patients at the Hub Center, but it presents challenges, such as the lack of direct patient contact and difficulties in accessing this resource for older patients and/or those from lower socioeconomic backgrounds [13]. Therefore, we believe that the referral back method can represent, especially in the field of LT, a valid and more equitable alternative to tele-consultation, as it allows for the continuation of adequate follow-up by expert professionals even outside the Hub hospital.

Similar projects have been described in the literature. In particular Tai et al. showed that Hub-and-spoke LT networks are effective in offering equivalent clinical outcomes, alleviating clinical pressure on the hub Centre, with high patient satisfaction [6]. Similarly, Tsien et al., highlighted delivering post-transplant patient care close to home is cost-effective without compromising patient survival and long-term clinical outcomes. [14]

Our project presents potential limitations. The first concerns the observation period (about four years), which is rather short considering the number of patients referred for evaluation. Moreover, it is possible that the referral of some patients was influenced by the ongoing COVID-19 pandemic. The second concerns the endpoints we decided to identify, especially regarding referral back and its impact on hospital activity, which we chose arbitrarily and, therefore, may be difficult to compare with other hospital settings. Moreover, this network may be most applicable in geographical areas where the distance between centers is relatively short, being difficult to put in place in different geographical areas.

We decided to focus only on the sickest patients (i.e., those hospitalized with acute decompensation, acute-on-chronic liver failure, or severe acute liver injury), as they could be homogeneously grouped in terms of short-term prognosis. These conditions have specific referral indications, which were applied in clinical practice [15–17]. Our study did not include patients referred as outpatients due to the heterogeneous indications for referral to the Hub Center. Additionally, we were unable to collect data on the entire pool of patients managed at the Spoke Center who were potentially eligible for referral. Therefore, our study may have excluded patients who, for various reasons, were not referred despite fulfilling the criteria. In the near future, we plan to create dedicated datasets to facilitate an intention-to-treat analysis.

Finally, we acknowledged that this was a pilot study without a control group. Looking at historical cohorts, a total of *n*. 5 and n. 7 inpatients from the Trentino Region were evaluated at Padua University Hospital between 2013–2016 and 2017–2019, respectively. Consequently, this integrated network led to a significant increase of referred patients (*n*. 27 patients in 3 years), significantly improving equity in access to LT. Additionally, the number of patients from Trento Hospital who achieved LT significantly increased (4 patients between 2013–2019 vs. 6 between 2020–2023). This suggests that the integrated referral process also contributed to increased access to transplantation.

In conclusion, this paper has described the referral and referral-back project for patients with complex end-stage liver disease, particularly those undergoing liver transplantation, as well as the follow-up after transplant. This experience could serve as a model for enhancing the network between Hub and Spoke Centers in managing complex liver disease cases and for addressing inequalities in the era of digital health.

## Data Availability

The data that support the findings of this study are available from the corresponding author upon reasonable request.
